# Evaluation of different teaching methods for FAST examination: high fidelity simulation versus traditional training

**DOI:** 10.1186/2036-7902-6-S2-A8

**Published:** 2014-08-27

**Authors:** L Astolfi, S Spadaro, G Zani, A Gioia, A Ferraresi, MV Colamussi, R Ragazzi, CA Volta

**Affiliations:** 1 Department of Morphology, Surgery and Experimental Medicine, Section of Anesthesia and Intensive Care, University of Ferrara, Ferrara, Italy

## Background

The Focused Assessment with Sonography for Trauma (FAST) is used to identify free fluids. It is highly sensitive and specific if performed by experienced physicians. The most complex part of the learning curve is represented by the acquisition of adequate images for diagnostic purposes. Hence it is of primary importance to properly handle the ultrasound probe and to identify its correct position and orientation on patients. High fidelity simulation (HFS) might provide an efficient way of learning by reproducing realistic and rare situations. However, although some ultrasound simulators are commercially available, none is of low cost, suitable for every medical discipline and specifically designed for HFS and made for any mannequin.

## Objective

The purpose of this study was to evaluate the use of an ultrasound simulator specifically designed for HFS during FAST exam.

## Patients and methods

This prospective study involved first- and second-year anesthesiology residents, without prior FAST training. Participants were randomly divided in two groups. The first group was trained by a standardized 2-hours didactic lecture about FAST exam, while the other was given 2-hours training using an innovative, versatile, low budget ultrasound simulator, specifically designed by our researchers. Both groups were trained by the same instructor and the same images were used. Then both groups practiced a FAST exam on a real multiple trauma patient. Four procedures were recorded: correct probe position, time to images acquisition, quality of images and their interpretation.

## Results

20 residents were enrolled, ten each group. All participants were able to obtain FAST images, but residents using the simulator were faster than those trained with the traditional lecture: 3.4 ± 0.7 and 4.3 ± 1.1 min, respectively (p<0.05). Although there were no relevant differences in terms of scores for images quality and interpretation, probably for the low number of resident enrolled, there was a tendency for the group trained in HFS to have better quality and interpretation of the acquired images.

## Conclusion

Medical training using simulation should have many advantages when compared to traditional training techniques. New low cost technologies applied to healthcare simulation make possible to create professional simulator to help learners in acquiring initial competence in FAST exam. However, further studies are needed to verify if this versatile ultrasound simulation has positive long term learning effects.

## References

[B1] Crouch AK Dawson M Long D Allred D Madsen T Perceived confidence in the FAST exam before and after an educational intervention in a developing country. International Journal of Emergency Medicine 2010 6 1 49 52 10.1007/s12245-009-0144-5 20414382 PMC2850974

[B2] Damewood S Jeanmonod D Cadigan B Comparison of a multimedia simulator to a human model for teaching FAST exam image interpretation and image acquisition. Academic Emergency Medicine 2011 6 4 413 419 10.1111/j.1553-2712.2011.01037.x 21496145

[B3] Parks AR Atkinson P Verheul G LeBlanc-Duchin D Critical Ultrasound Journal 2013 6 1 9 9 10.1186/2036-7902-5-9 24245514 PMC3843513

